# Computer grading of lung disease severity in patients with lymphangioleiomyomatosis referred for transplantation

**DOI:** 10.1186/s12890-022-02123-7

**Published:** 2022-09-24

**Authors:** Angelo M. Taveira-DaSilva, Vissaagan Gopalakrishnan, Jianhua Yao, Marcus Y. Chen, Patricia Julien-Williams, Amanda M. Jones, Gustavo Pacheco-Rodriguez, Joel Moss

**Affiliations:** 1 Pulmonary Branch, NHLBI, NIH, Building 10, Room 6D05, MSC 1590, Bethesda, MD 20892-1590 USA; 2 Radiology and Imaging Sciences Department, NIH, Bethesda, MD 20892 USA; 3 Cardiovascular Branch, NHLBI, NIH, Bethesda, MD 20892 USA

**Keywords:** Lymphangioleiomyomatosis (LAM), Lung transplant, Cystic lung disease, High resolution computed tomography (HRCT)

## Abstract

**Objectives:**

Lymphangioleiomyomatosis (LAM) patients with severe lung disease may be considered for lung transplantation. Clinical, physiologic, and quality of life data are usually employed for referral. The aim of this study was to determine whether computed tomographic measurement of lung volume occupied by cysts (cyst score) complemented clinical and physiologic data in supporting referral for transplantation.

**Methods:**

Forty-one patients were studied. Pre-referral clinical data, pulmonary function tests, exercise testing, and high-resolution computed tomography (HRCT) scans were obtained. From HRCT, a computer-aided diagnostic program was employed to calculate cyst scores. These data were compared to those of 41 age-matched LAM patients not referred for lung transplantation.

**Results:**

Cyst score, and % predicted FEV_1_ and DL_CO_ were respectively, 48.1 ± 9.4%, 36.5 ± 9.1%, and 35.0 ± 10.7%. For the control group, cyst score, FEV_1_, and DL_CO_ were respectively, 14.8 ± 8.3%, 77.2 ± 20.3%, and 66.7 ± 19.3%. Cyst score values showed a normal distribution. However, the frequency distribution of FEV_1_ was skewed to the right while the distribution of DL_CO_ was bimodal. Correlations between cyst score and FEV_1_ and DL_CO_ for the study group were respectively, r = − 0.319 and r = − 0.421.

**Conclusions:**

LAM patients referred for lung transplantation had nearly 50% of lungs occupied by cysts. Correlations between cyst score and FEV_1_ or DL_CO_ were weak; as shown previously, DL_CO_ was better related to cyst number while FEV_1_ had a better association with cyst size. Given its normal distribution, cyst score measurements may assist in evaluation of pre-transplant severity of lung disease before referral for transplantation.

**Supplementary Information:**

The online version contains supplementary material available at 10.1186/s12890-022-02123-7.

## Background

Lymphangioleiomyomatosis (LAM), a multi-system disease that primarily affects women, is characterized by cystic lung destruction, lymphatic involvement (e.g., chylous effusions, lymphangioleiomyomas), and abdominal tumors, e.g., angiomyolipomas (AML) [1–3]. LAM occurs sporadically or in association with Tuberous Sclerosis Complex (TSC), an inherited disorder associated with seizures, cognitive impairment, and dermatological manifestations [4]. LAM lung destruction results from the presence of abnormal LAM cells, which have both melanocyte and smooth muscle cell characteristics and harbor mutations of the *TSC1* or *TSC2* genes [5–7]. The loss of function of two proteins, hamartin and tuberin, encoded respectively by *TSC1* and *TSC2* genes, results in activation of the mechanistic Target of Rapamycin (mTOR) signaling pathway, which is responsible for cell growth, size, and survival [8].

The course of lung disease in LAM is highly variable but may result in progressive loss of lung function, severe exercise limitation, and respiratory failure, requiring transplantation [1, 2]. Studies showed that sirolimus therapy stabilized lung function, improved symptoms and quality of life [9], reduced size of AML [10], and achieved resolution of lymphatic tumors and chylous effusions [11]. For those patients with severe disease who fail to respond to mTOR inhibitor therapy, lung transplantation becomes a strong consideration [2, 12].

Precise clinical and physiologic criteria for lung transplantation in LAM have not been established. In contrast to some other lung diseases, e.g., interstitial pulmonary fibrosis, pulmonary hypertension, patients with LAM, even those requiring supplemental oxygen, are frequently comfortable at rest. Consequently, criteria based on clinical data, pulmonary function tests, exercise testing, and oxygen requirements, are usually employed [12–15]. Analysis of data from LAM patients prior to lung transplantation show that most patients had FEV_1_ and DL_CO_ under 30% predicted [12–14]. Consequently, some have suggested that FEV_1_ and DL_CO_ values near 30% predicted, are an indication for evaluation for transplantation [2, 12–14]. However, these criteria have not been validated [12–14].

Grading of disease severity and monitoring of disease progression in LAM are frequently accomplished by examination of the patient, serial measurements of lung function, and imaging studies [2, 13]. These tests, however, may require patient cooperation, and may fail to uncover gas exchange abnormalities that become manifest only during exercise. Accordingly, because of these limitations, those tests are usually complemented by cardiopulmonary exercise testing [2, 13, 15].

High-resolution computed tomography (HRCT) scans of the chest are used in the diagnosis and evaluation of LAM [2, 13]. Radiologic studies, including calculations of a disease-specific radiologic score, determination of areas with lower attenuation and texture, or calculation of the percent of lung volume occupied by cysts (cyst score) have been reported to correlate with physiologic data [16–20].

The aim of this study was to grade the severity of disease by a computer-based computer tomography technique and correlate it with clinical and physiologic data in LAM patients with severe symptomatology who were either referred for lung transplantation or had undergone lung transplantation. The objective was to determine whether HRCT imaging data would complement physiologic and clinical data in supporting a recommendation for lung transplantation.

## Methods

Forty-one patients are the subject of this study. Thirty-three of the 41 patients had been advised to undergo pre-transplant evaluation at several transplantation centers. The remaining eight patients had undergone lung transplantation. Nineteen patients did not complete evaluation. Two died who were waiting evaluation. Patients were selected for transplantation evaluation based on clinical data including use of oxygen, intolerance to activities of daily living, and the presence of severe disease by pulmonary function and computer tomography imaging. Once this cohort was identified, we selected a second group of 41 patients from our database who matched the cohort patients by age but had not been referred for transplantation evaluation. An essential requirement was that both the study group and control group had undergone computer tomography imaging that was suitable for measurement of cyst score.

Patients were diagnosed with LAM based on American Thoracic Society/European Respiratory Society guidelines [21, 22]. History, physical examination, pulmonary function tests, and computed tomography studies were obtained. The study was approved by the National Heart, Lung, and Blood Institute Institutional Review Board. All patients participated in LAM natural history and pathogenesis protocols (NHLBI Protocols 95-H-0186 and 96-H-0100), and provided written informed consent before enrollment and all methods were performed in accordance with the ethical guidelines and regulations.

### Pulmonary function

Lung volumes, flow rates, and DL_CO_ were measured using a Vmax Encore (Vyaire Medical, Yorba Linda, CA, USA) system, per American Thoracic Society/European Respiratory Society standards [23, 24]. In most of the patients, testing was performed on the same hospital visit as the CT examination.

### Cardiopulmonary exercise testing

Patients exercised on a bicycle ergometer and used a computerized metabolic cart (Vmax 229 Cardiopulmonary Exercise System; Sensormedics, Yorba Linda, CA, USA), according to standard incremental protocols [15].

### Six-minute walk test

Six-minute walk tests were performed according to the European Respiratory and American Thoracic Societies guidelines [25].

### Radiologic methods

HRCT scans of the chest (GE Medical Systems, Waukesha, WI; Phillips Healthcare, Amsterdam, Netherlands; and Siemens Healthcare, Erlangen, Germany) were performed with the patients in a prone position during end inspiration at 120–140 kilovolt peak and 118–560 mA, with 1- to 2-s scanning times and 34- to 38-cm field of view [16–19]. Scans contained 9–13 slices, with slice thickness ranging from 1 to 1.25 mm at 3-cm intervals. Images were reconstructed using a lung kernel (filter) to emphasize the range of pixel values for lung tissues.

### Lung segmentation and cyst detection

Cyst score is calculated based on the ratio of low-attenuation cyst volume to total lung volume. A computer-aided diagnostic system, built using C++ and MATLAB (MathWorks, Natick, MA), segments the lungs and classifies pixel blocks of tissue as cystic or non-cystic, based on image texture and threshold values (see Additional file 1). Lung segmentation is a multi-step process that first identifies lung boundaries and then excludes the trachea and blood vessels. Segmented lung regions are then divided into pixel blocks, which undergo classification as cystic or non-cystic using a committee of support-vector machines trained with radiologist input [17]. Cystic regions and total lung areas are calculated for every slice in a CT examination, summed separately, and then divided, yielding a total percent of lung volume occupied by cysts (cyst score) [16–18].

This technique represents an improvement from prior qualitative radiologic grading of LAM disease severity (mild, moderate, and severe), and has been validated in multiple other studies, showing strong correlations with pulmonary function tests in cohorts of patients with varying extent of disease [17–19] and tracking with LAM disease progression over time in individual patients [18]. An online supplement contains more specifics regarding the calculation technique.

### Analysis of data

The correlation between cyst score and FEV_1_ and DL_CO_ was evaluated by the Pearson correlation method. A control group of 41 patients, age-matched with the cohort referred for transplantation, were compared to the study group.

## Results

### Demographics

Thirty-seven of the 41 patients were White, two were African-Americans and two Asian-Americans. Five had TSC-LAM and 36 had sporadic LAM. Symptoms at the time of diagnosis were dyspnea in 21, pneumothorax in 15, hemoptysis in four, and pleural effusion in one (Table 1). Age of diagnosis for all patients was 42.5 ± 11.4 years. First symptoms occurred at the age of 39.0 ± 10.4 years. The mean age of the patients at the time of data collection was 54.9 ± 12.5 years. Patients had the initial evaluation prior to the establishment of sirolimus as treatment for patients with LAM; therefore, the conclusions reflect that subgroup of patients. The age of the eight patients who underwent lung transplantation was 46.3 ± 5.4 years. Pulmonary function data for this cohort are shown in Table 2. The mean follow-up time prior to enrollment was 9.9 ± 6 years.

**Table 1:** Demographic data from 41 LAM patients referred for lung transplantation

Number of patients	41
Age at time of study	54.9 ± 12.5 years
Age of LAM diagnosis	42.5 ± 11.4 years
Age of first symptoms	39.0 ± 10.4 years
*Initial presentation*	
Dyspnea	21
Pneumothorax	15
Hemoptysis	4
Pleural effusion	1
Cough	2
Chest pain	1
Abdominal pain	1
No symptoms	1
*Mode of diagnosis*	
Lung biopsy	21
Abdominal mass biopsy	2
Characteristic lung CT findings and TSC	5
Characteristic lung CT findings and lymphangioleiomyoma	8
Characteristic lung CT findings and angiomyolipoma	2
Characteristic lung CT findings and elevated serum VEGF-D levels	3

**Table 2:** Pulmonary function tests of 41 LAM patients before referral for lung transplantation

TLC (liters)	4.8 ± 1.2
TLC (% predicted)	96.0 ± 19
FRC (liters)	3.2 ± 0.9
FRC (% predicted)	115.2 ± 29.3
RV (liters)	2.3 ± 0.8
RV (% predicted)	126.9 ± 43.9
RV/TLC ratio (%)	47 ± 9
FVC (liters)	2.4 ± 0.8
FVC (% predicted)	75.5 ± 19.8
FEV_1_ (liters)	0.88 ± 0.25
FEV_1_ (% predicted)	36.4 ± 9.1
FEV_1_/FVC ratio (%)	34 ± 10
DL_CO_ (ml/min/mmHg/)	6.9 ± 2.1
DL_CO_ (% predicted)	34.8 ± 10.8

*DL*_*CO*_ diffusion capacity for carbon monoxide, *FEV*_*1*_ forced expiratory volume in one second, *FRC* functional residual capacity, *FVC* forced vital capacity, *RV* residual volume, *TLC* total lung capacity

The age of the control group was 54.0 ± 12.7 years. Thirty-four patients were White, three African-American, three Asian and one Hispanic. Their age at the time of the first symptoms was 39.2 ± 9.9 years and the age at the time of diagnosis was 42.4 ± 10.5. Twenty-seven patients had dyspnea, 13 had pneumothorax, five had hemoptysis, one had bleeding from an angiomyolipoma, and another was asymptomatic. Twenty-one patients of the control group were diagnosed by tissue biopsy, nine had angiomyolipomas, two had TSC, two had chylous effusions, one had an elevated VEGF-D level, and six had extra-pulmonary LAM.

### Prevalence of pneumothorax: chylothorax and use of supplemental oxygen on transplant and control groups

Of the 41 transplantation group patients, 20 had history of pneumothoraces of which 15 were bilateral. Seven patients had chylothorax. There was no significant difference between this group and the control group. In the control group, there was a total of 17 pneumothoraces of which seven were bilateral, and six chylous effusions (see Additional file 2: Table S1). There was a significant difference between the transplant and control group regarding the number of patients on continuous supplemental oxygen, 39 versus 9.

### Cyst scores, FEV_1_ and DLCO

**Fig. 1:**
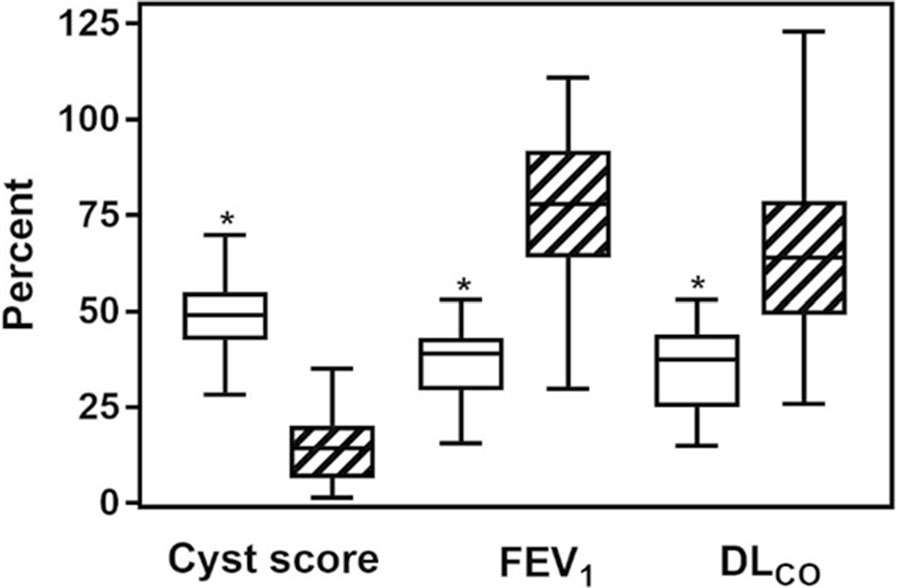
Box-and whiskers plot showing percent cyst score, and corresponding percent predicted FEV_1_ and DL_CO_ from 41 LAM patients who were referred for or underwent lung transplantation (white bars), and a group of 41 patients matched by age, who were not referred for transplantation evaluation (hatched bars). The top line represents the 75% quartile and the lower line represents the 25% quartile. The line across the boxes represents the second quartile (median). The upper and lower whiskers indicate the maximum and minimum values. The patients who were referred for or underwent lung transplantation have significantly higher cyst score and lower FEV_1_ and DL_CO_, *p < 0.001

**Fig. 2:**
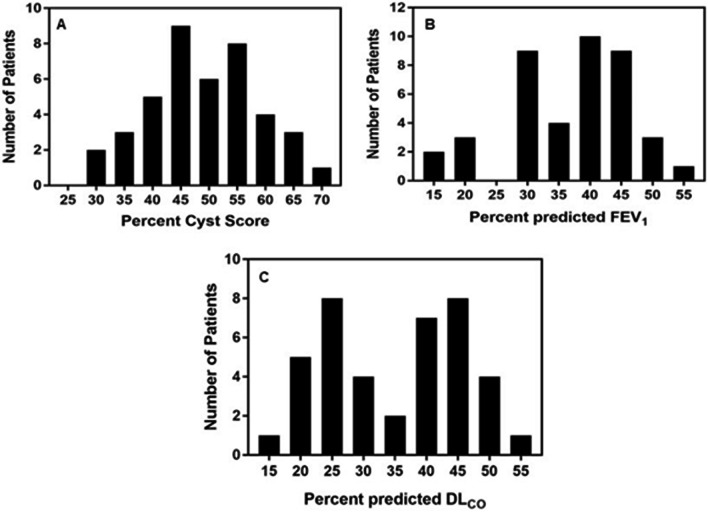
Panels **A**–**C**. Frequency distribution of cyst scores (**A**), FEV_1_ (**B**), and DL_CO_ (**C**), in 41 LAM patients with severe LAM. The frequency distribution of cyst scores is normal. Note that the frequency distribution of the FEV_1_ is skewed to the left and the frequency distribution of the DL_CO_, is bimodal

The mean cyst score was 48.6 ± 9.6%. The median value was 49.1% (Table 3, and Fig. 1). Cyst score values for those patients who had been transplanted, prior to transplantation, were very similar, 47.7 ± 9.4%. Mean percent-predicted FEV_1_ and DL_CO_ for the 41 patients were 36.4 ± 9.1 and 34.8 ± 10.8%, respectively (Table 3, Fig. 1). For the patients who were subsequently transplanted, mean percent-predicted FEV_1_ and DL_CO_ were respectively, 29.6 ± 10.1 and 32.7 ± 13.6%. Cyst score distribution for the 41 patients is shown on Fig. 2, panel A. The distribution of percent-predicted FEV_1_ and DL_CO_ is shown on Fig. 2, panels B, and C.

**Table 3:** Percent cyst score and percent predicted FEV_1,_ and DL_CO_ of 41 LAM patients referred for lung transplantation

	% Cyst score	% FEV_1_	% DL_CO_
Mean	48.6	36.4	34.8
Median	49.1	38.9	37.5
25% percentile	42.5	29.3	25.0
75% percentile	54.9	43.1	44.0
Minimum	28.2	15.6	15.0
Maximum	69.9	53.1	53.0

The median time between measurement of the cyst scores and pulmonary function tests was 0.0 ± 0.0 months. The interquartile range was 5.28 months.

**Fig. 3:**
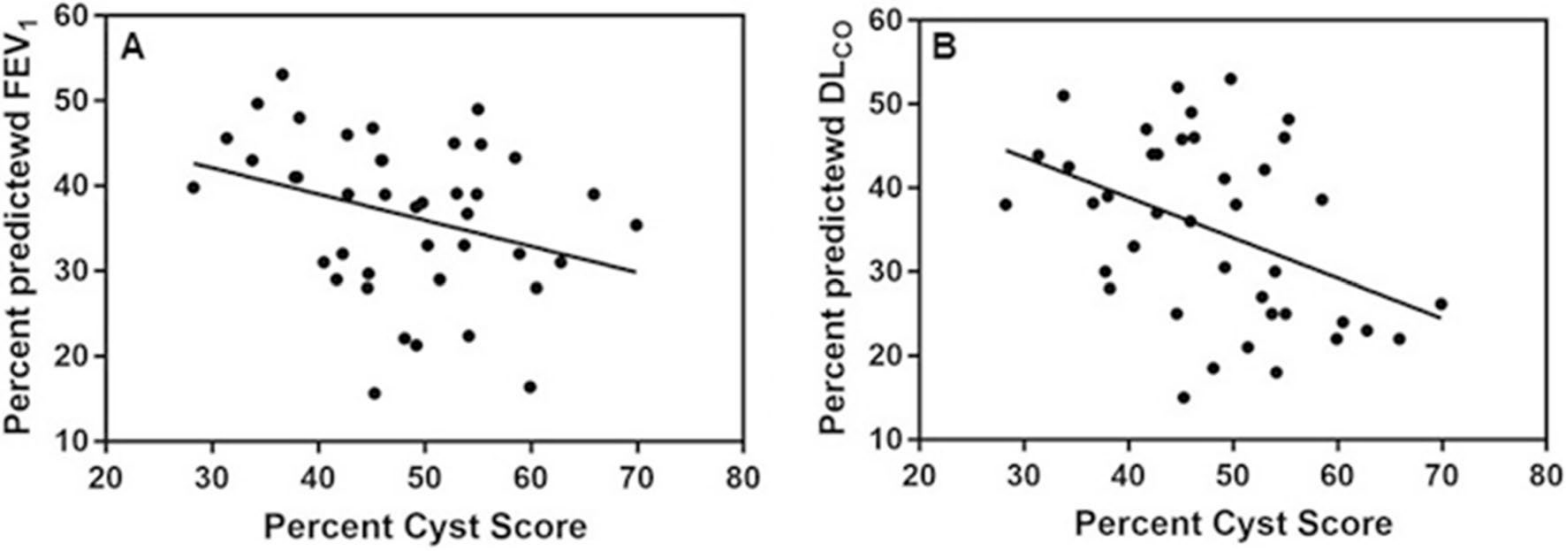
Panels **A**, **B**. Relationship between cyst score and FEV_1_ (**A**), and DL_CO_ (**B**), in 41 LAM patients with severe lung disease referred for lung transplantation

The correlation between cyst score and FEV_1_ was r = − 0.357, *p* = 0.021. For the DL_CO_, the correlation was r = − 0.447, *p* = 0.003 (Fig. 3). The correlation between FEV_1_ and DL_CO_ was r = 0.502, *p* < 0.001.

Cyst score, and percent-predicted FEV_1_ and DL_CO_ for the LAM patient control group were respectively, 14.4 ± 8.8, 77.0 ± 19.9, and 65.3 ± 20.8% (Fig. 1). These values are significantly different from those of the transplantation cohort (*p* < 0.001).

### Cardiopulmonary exercise tests and six-minute walk tests

Twenty-seven patients underwent an incremental bicycle exercise test within a year of having the CT scans and pulmonary function tests. Five patients exercised while receiving supplemental oxygen and the remaining 22 patients exercised on room air. Peak heart rate, 83 ± 11%, oxygen pulse, 77 ± 17%, peak oxygen uptake (VO_2_), 64 ± 16%, and work-load, 77 ± 23% were reduced. Oxygen saturation decreased from 97 ± 5 to 92 ± 5% (Table 4).

**Table 4:** Cardiopulmonary exercise and six-minute walk tests in 40 patients with LAM referred for lung transplantation

*Cardiopulmonary exercise test*	
Number subjects	27 (% predicted)
Peak heart rate (beats/min)	145 ± 24 (83 ± 11%)
O2 pulse (ml/beat)	7.3 ± 2.1 (77 ± 17%)
Breathing reserve	24.6 ± 17.3
VECO2/AT	41
Load (watts)	95 ± 34 (77 ± 23%)
Peak VO2 (ml/min)	1075 ± 406 (64 ± 16%)
Resting SaO2 (%)	96.7 ± 2.6
Peak SaO2 (%)	92.3 ± 2.6
Change SaO2 (%)	4.3 ± 3.3
*Six minute walk test*	
Number subjects	13
Resting heart rate (beats/min)	88 ± 10
Peak heart rate (beats/min)	119 ± 20
Supplemental O2 (l/min)	1.1 ± 2
Distance walked (meters)	395 ± 120
Resting SaO2 (%)	97 ± 2.5
Peak SaO2 (%)	86.8 ± 6.1
Resting dyspnea index (Borg units)	0.7 ± 0.8
Peak dyspnea index (Borg units)	5.2 ± 2.4

Data are shown as means ± SD

*HR* heart rate, *VO2 max* peak oxygen uptake, *VO2 max/HR* oxygen pulse, V*E max* minute ventilation at peak exercise, *BR* breathing reserve, *VE/VCO2 AT* ventilatory equivalent for carbon dioxide at anaerobic threshold, *SaO2* pulse oxygen saturation (%)

Thirteen patients who could not perform CPET underwent six-minute walk testing, four on supplemental oxygen and nine on room air. The average distance walked was 395 ± 120 m. Peak heart rate was 119 ± 19 beats/min. Oxygen saturation fell on all patients, including those receiving supplemental oxygen, from 97 ± 2 to 87 ± 6% (see Table 4).

### Outcome

A total of eight patients underwent lung transplantation. Of these, three patients are alive 14.6 ± 0.5 years after transplantation. The remaining five patients expired within 5.3 ± 4.6 years after transplantation. Of the remaining 33 patients two died before transplantation, 12 underwent pre-transplantation testing, and 19 had not yet undergone pre-transplantation testing. The cause of death of the patients who were not transplanted was lung disease.

## Discussion

Our study shows that LAM patients who are referred for or undergo lung transplantation have cyst scores averaging near 50%. That is, one half of the lung volume comprises cystic lesions that can be detected by current computed tomography imaging techniques. The remaining lung parenchyma however, may comprise both normal lung or areas containing small cystic lesions that were not detected by the current technique. Hence, the percentage of lung parenchyma ocupied by cysts could even be greater than 50%. In contrast to these findings, the control age-matched patient population had a mean cyst score of 14.8 ± 8.3% and an FEV_1_ and DL_CO_ of 77.2 ± 20.3 and 66.7 ± 19.3% predicted, respectively (Fig. 1).

**Fig. 4:**
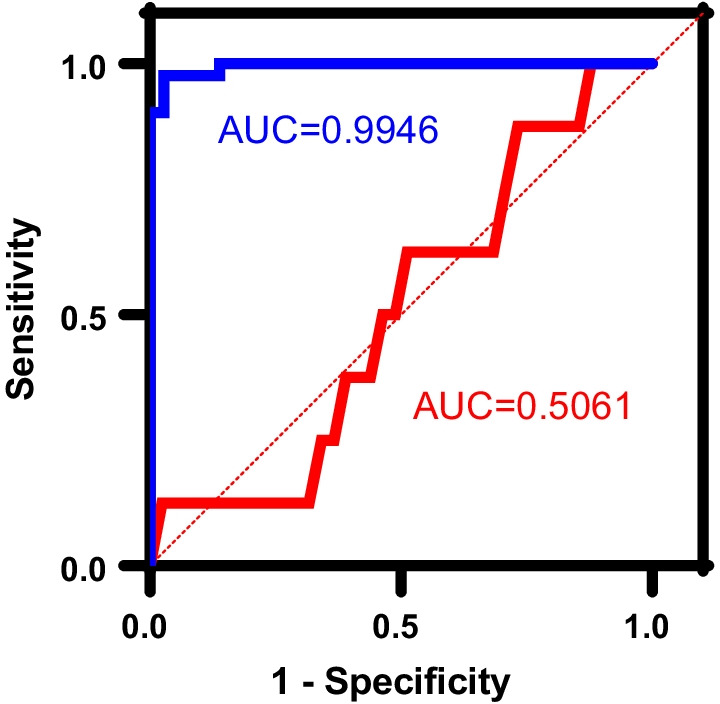
A ROC curve of the percent cyst score from patients that could be defined to be referred for lung transplantation based on the data from both the study group and an age-based control group with less severe disease. Transplanted group, red line; control group, blue line

Correlations between cyst score and FEV_1_ and DL_CO_, were relatively low. This is, in part, because although the frequency distribution of cyst scores in our population appeared to be normal, the same was not the case for the FEV_1_ and especially for the DL_CO_. The distribution of FEV_1_ was skewed to the right whereas the distribution of DL_CO_ was bimodal. In fact, reductions in FEV_1_ did not parallel reductions in DL_CO_. That is, some patients had either an FEV_1_ or a DL_CO_ above 50% predicted, which by itself would not be considered an indication for lung transplantation. Indeed, in a recent study, we reported that out of 84 pre-menopausal LAM patients, only 29 (34%), had both FEV_1_ and DL_CO_ values ≤ 70% predicted [26]. This is consistent with observations that some LAM patients present with mild impairment of flow rates, e.g., FEV_1_, and an isolated reduction in DL_CO_. Some phenotypes, characterized by numerous small cysts tend to be associated with greater impairment in gas exchange than lung mechanics [27]. A recent study, showed that ultra-small cysts primarily contribute to the reduction in DLco, with minimal effects on FEV_1_ [28]. Patients with lower cyst burden and better FEV_1_ tended to have smaller average cyst size and higher ultra-small cyst fraction [28]. Figure 4 shows ROC curves for control (% Cyst score) and all LAM patients (blue curve) and the control group and show that there is almost a perfect match of patient not requiring referred for transplantation (sensitivity). The area under the curve (AUC) shows that the correlation is almost 1. On the other hand, when we graph all LAM patients and transplanted patients (red line), the Figure shows that the chances that we can assign a patient to the transplanted base is almost 50%.

Conversely, other patients have markedly reduced FEV_1_, with CT scans showing larger cysts and with a well-preserved DL_CO_ [27]. This mismatch could explain the poor correlation between FEV_1_ and DL_CO_ (r = 0.502) in the transplant cohort studied here. We suggest that patients with larger cysts, who reportedly have a greater rate of decline in FEV_1_ than those with smaller cysts [27, 28], may have relatively normal lung parenchyma between the cysts, causing lung diffusion capacity to be relatively normal. Another factor affecting the correlation between cyst scores and lung function is that small lung cysts may not be detected by the computer-based technique employed in this study, but require using techniqes that detect ultra-small cysts, that especially impairf lung diffusion capacity [28].

Our findings are consistent with those of three prior studies [18–20]. Yao et al. [18], using similar imaging techniques reported that cyst scores stabilized during sirolimus therapy and this effect was associated with stabilization of lung function. Since all the patients evaluated here were not receiving sirolimus treatment,our conclusions should reflect that subgroup of patients. Evidence of stabilization in rate of change of cyst scores suggested that sirolimus therapy may reduce cyst size or formation of new cysts. Argula et al. [20] used a watershed algorithm to assess changes in cystic lesions in a subgroup of patients who participated in the MILES trial. They found that the number of cysts at residual volume increased in the placebo group and this was associated with worsening of air trapping. Gopalakrishnan et al. [19], reported that treatment with sirolimus stabilized cyst score and improved lung texture in areas surrounding the cysts These studies, altogether, suggest that cyst volumetric analysis is an important tool that complements physiologic testing. Indeed, exercise testing, which complements standard pulmonary function tests by uncovering what may be occult hypoxemia, showed that patients referred for transplantation had reduced exercise capacity, and experienced arterial de-saturation during exercise (Table 4) [15].

Indications for lung transplantation evaluation in patients with LAM have not been precisely established. This is in part due to the fact that the severity of symptoms in patients with LAM at rest is highly variable and lung transplantation still has significant morbidity, expenses, and relatively low long-term survival [14, 29]. A study reported data from 12 LAM patients (age, 42 ± 8 years) who underwent lung transplantation [29]. Their mean percent predicted FEV_1_ and DL_CO_ were respectively 19 ± 11, and 23.6 ± 9%. Average six-minute walk test distance was 273 ± 117 m, and all patients had exercise-induced hypoxemia. Eight patients had pulmonary hypertension. A study from Japan [14] conducted in 57 transplanted patients reported survivals of 86.7% at one year, 82.5% at three years, 73.7% at five years, and 73.7% at 10 years. However, when the 57 transplanted patients were compared with 41 patients referred for, but not transplanted, no statistically significant difference in post-registration survival between the transplanted group and the waiting group was observed: 94.7% survived at one year, 91.1% at three years, 84.9% at five years, and 73.0% at 10 years, versus 91.8%, 75.6%, 75.6%, and 33.6%, respectively, for the non-transplanted patients. Percent-predicted FEV_1_ and DLco in the transplanted group of patients were respectively 32.8 ± 17.0% (range = 9.1–77.5) and 24.7 ± 11.2% (range = 0.7–58.3), a broad range of values. In most studies reported in the literature, pre-transplant lung function and six-minute walk test data were respectively 24–33% predicted for the FEV_1_, 23–38% predicted for the DL_CO_ and a six-minute walk test distance under 250 m [12, 30–34]. This wide range of data confirms that lung function tests alone may not accurately determine the precise timing for transplantation evaluation.

We suggest that, in contrast to FEV_1_ or DL_CO_, cyst score data seemed to have a more normal distribution because they measure the percentage of lung parenchyma occupied by cysts regardless of their size and not the severity of air flow obstruction, which may or may not be related to the number of cysts. As the accuracy and precision of its measurement improves, especially the contribution to the severity of ultra-small cysts to the severity of disease, quantification of cystic lung disease may be a better measure of severity of lung disease than either FEV_1_ or DL_CO_, and add important new information in assisting with the evaluation of LAM patients for referral for lung transplantation [28].

## Conclusions

Based on these findings, we suggest that patients on continuous supplemental oxygen, and who experience hypoxemia during exercise, have an FEV_1_ and DL_CO_ around 30% predicted and cyst scores employing ultra-small cysts techniques [28]. At or above 50%, be considered for evaluation for lung transplantation. The presence of pulmonary hypertension strengthens a recommendation for such a referral [35, 36]. Patients must be informed about long-term lung transplantation survival rates in LAM, and advised to consider lung transplantation if they judge their quality of life to be poor. Clinical, physiologic, and imaging data assist in making such a decision, but are not the most important criteria for transplantation referral [2, 15]. Patient’s quality of life and willingness to consider having the procedure must be assessed prior to initiating such an evaluation. As imaging technology improves, measurement of cyst scores may serve as another useful method for grading lung disease severity in LAM patients being considered for transplantation.

## Supplementary Information

**Additional file 1.** Online Supplement on LAM Computer-Aided Diagnostic System.

**Additional file 2.** Comparison of Clinical, Functional, and Cyst Score (%) between Patients referred for Lung Transplantation (n=41).

## Data Availability

The datasets used and/or analysed during the current study are availiable upon request by the corresponding author.
