# Supplementary material for: Computer grading of lung disease severity in patients with lymphangioleiomyomatosis referred for transplantation
Source: BMC Pulm Med. 2022 Sep 24;22:362. doi: 10.1186/s12890-022-02123-7 (PMC9509628; doi:10.1186/s12890-022-02123-7)
Supplement: Supplementary file 1 — Additional file 1. Online Supplement on LAM Computer-Aided Diagnostic System. [file 12890_2022_2123_MOESM1_ESM.docx]

**On-line Supplement 1**

**Information on the LAM Computer-Aided Diagnostic System**

The goal of the computer-aided diagnostic (CAE) system was to provide more information regarding the extent of lung involvement in Lymphangioleiomyomatosis. Prior to the development of the CAD system, radiologists would provide grading of lung disease severity based on a highly qualitative assessment of HRCT scans (grades 0-3, corresponding to none, 0-30% lung involvement, 30-60% lung involvement, and 60-90% lung involvement). This method was subjective and lacked precision.

The in-house developed software was built using MATLAB and C++. The initial training of the computer model to classify pathologic versus non-pathologic tissue required input from a radiologist. Lung segmentation relies on the grayscale contrast between the lungs and surrounding tissues, while the identification of cystic versus non-cystic regions incorporates texture patterns of the lung tissue.

The general steps for the system are shown below:


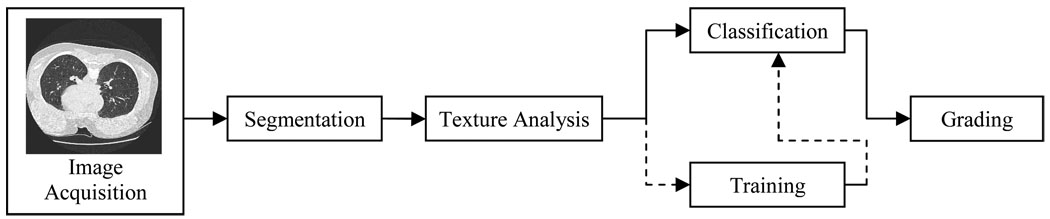


**Supplemental Figure 1. Steps to determine CT grading.**

***Lung Segmentation****.* First, the trachea is located and segmented out. By using the segmented position of the trachea, two initial seed points are assigned in the right and left lung. A 2D/3D recursive region growing algorithm (MATLAB) is then used to expand the segments to the entire lung. A histogram-based threshold technique removes blood vessels and a rolling ball algorithm is applied to remove background and smooth/refine the lung boundary. Lung volume is then calculated separately for the left and right lung.


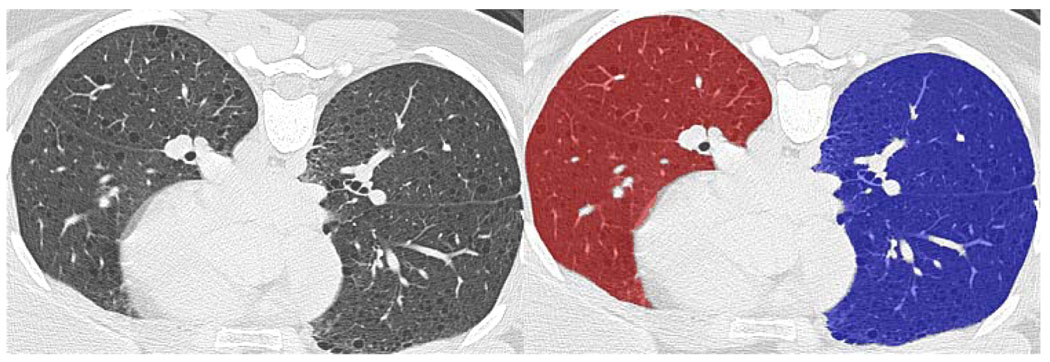


**Supplemental Figure 2. Left CT of the lungs and the right shows the transformation for the calculation.**

***Texture Analysis****.* Segmented lung regions are further subdivided into 16x16 pixel texture blocks, on which a multi-dimensional feature vector is computed. The feature vector is composed of 25 texture properties.

***Training***. In the initial training process, a radiologist manually selected portions of the lung within cysts (LAM) and outside of cysts (normal). Texture features were calculated for all regions and input to a support vector machine that dynamically selected attributes to distinguish cystic and non-cystic regions.

***Classification*.** A histogram of the lung region is computed and the threshold value for normal lung tissue (identified based on parameters set by the radiologist) is calculated using a K-means clustering algorithm. This thresholding is used to classify individual pixel blocks as cystic or non-cystic.


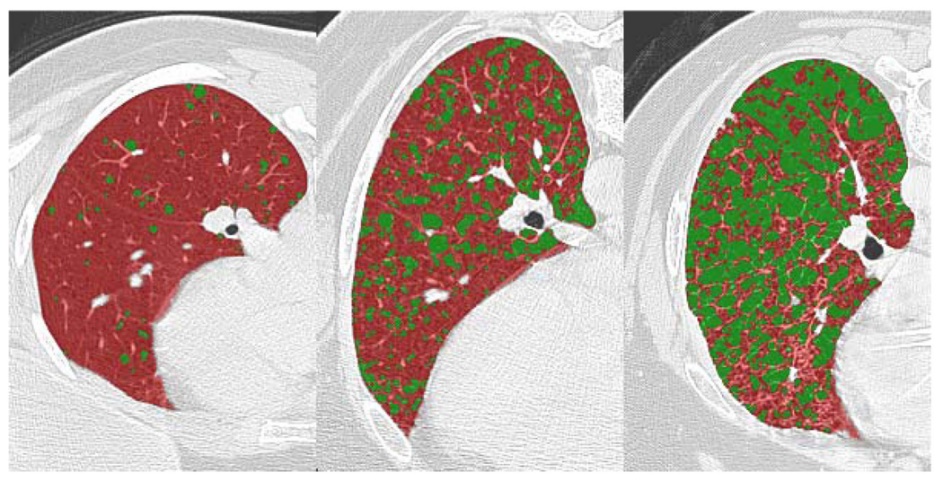


***Supplemental Figure 3. Images to calculate the cyst score.***

***Grading*.** ‘Cyst Score’ is calculated as the percentage of lung volume occupied by cysts.

This LAM CAD system was first presented at the 19th International Conference on Pattern Recognition in 2008 with a test set of 36 patients showed a strong correlation to subjective radiologist grading (R=0.91, *p*<0.0001). It has since been further validated in numerous LAM patient cohorts.

Proc IAPR Int Conf Pattern Recogn Abstract: [https://www.ncbi.nlm.nih.gov/pubmed/21625320](about:blank)
